# Epidermal Growth Factor Receptor (EGFR)-RAS Signaling Pathway in Penile Squamous Cell Carcinoma

**DOI:** 10.1371/journal.pone.0062175

**Published:** 2013-04-24

**Authors:** Hong-Feng Gou, Xiang Li, Meng Qiu, Ke Cheng, Long-Hao Li, Hang Dong, Ye Chen, Yuan Tang, Feng Gao, Feng Zhao, Hai-Tao Men, Jun Ge, Jing-Mei Su, Feng Xu, Feng Bi, Jian-Jun Gao, Ji-Yan Liu

**Affiliations:** 1 Department of Medical Oncology, Cancer Center, The State Key Laboratory of Biotherapy, West China Hospital, West China Medical School, Sichuan University, Chengdu, China; 2 Department of Urology, West China Hospital, West China Medical School, Sichuan University, Chengdu, China; 3 Department of Pathology, West China Hospital, West China Medical School, Sichuan University, Chengdu, China; 4 Department of Genitourinary Medical Oncology, The University of Texas M. D. Anderson Cancer Center, Houston, Texas, United States of America; University Magna Graecia, Italy

## Abstract

Penile Squamous Cell Carcinoma (SCC) is a rare cancer with poor prognosis and limited response to conventional chemotherapy. The genetic and epigenetic alterations of Epidermal Growth Factor Receptor (EGFR)-RAS-RAF signaling in penile SCC are unclear. This study aims to investigate four key members of this pathway in penile SCC. We examined the expression of EGFR and RAS-association domain family 1 A (RASSF1A) as well as the mutation status of K-RAS and BRAF in 150 cases of penile SCC. EGFR and RASSF1A expression was evaluated by immunohistochemistry. KRAS mutations at codons 12 and 13, and the BRAF mutation at codon 600 were analyzed on DNA isolated from formalin fixed paraffin embedded tissues by direct genomic sequencing. EGFR expression was positive in all specimens, and its over-expression rate was 92%. RASSF1A expression rate was only 3.42%. Significant correlation was not found between the expression of EGFR or RASSF1A and tumor grade, pT stage or lymph node metastases. The detection of KRAS and BRAF mutations analysis was performed in 94 and 83 tumor tissues, respectively. We found KRAS mutation in only one sample and found no BRAF V600E point mutation. In summary, we found over-expression of EGFR in the majority cases of penile SCC, but only rare expression of RASSF1A, rare KRAS mutation, and no BRAF mutation in penile SCC. These data suggest that anti-EGFR agents may be potentially considered as therapeutic options in penile SCC.

## Introduction

Penile squamous cell carcinoma (SCC) is a relatively rare disease and accounts for less than 1% of all male malignancies in Europe and North America [1]. Its incidence is significantly higher in under-developed countries. In China, the incidence of penile SCC has also been declining gradually over the past several decades due to continuous improvement of health care conditions. Because of its low incidence, penile SCC treatments have been rarely studied and reported in the literature. Surgery is the first choice for localized, resectable penile SCC. However, surgery is destructive, and more than half of the patients will recur or metastasize within 5 years even after radical resection. For advanced diseases, palliative surgery and radiation therapy may be considered for local disease control and prevention of complications, partly due to lack of effective drugs for the disease. Chemotherapeutic agents showed limited effectiveness with a short-term response rate of less than 30% and a 3-year survival rate of less than 10% for metastatic penile cancer [2], [3]. Thus, there is an urgent need to develop new treatment strategies for penile SCC. Recently, target therapies showed promising anticancer activities in a various types of cancer. However, little work has been done to evaluate their effectiveness in penile SCC. Therefore, elucidation of the molecular pathways involved in penile SCC is essential for understanding the pathogenesis of and developing new treatment strategies for this rare disease.

The epidermal growth factor receptor (EGFR)-RAS-RAF signaling pathway plays an important role in regulation of tumor cell survival and proliferation. EGFR is highly expressed in a variety of epithelial tumors, such as non-small cell lung cancer, head and neck squamous cell carcinoma (HNSCC), colorectal cancer (CRC), and breast cancer [4], [5]. Multiple anti-EGFR agents have been developed and have exhibited significant anti-tumor activities in these cancers [6], [7].

The KRAS gene, a member of the ras proto-oncogene family, encodes a protein that is an important component of the EGFR signaling pathway. KRAS mutations are linked to a poor response to EGFR inhibition and resistance to anti-EGFR agents [8]. KRAS mutations are mostly found in codons 12 and 13 (exon 2), and occasionally in codon 61 (exon 3). KRAS mutations frequency varies in different human tumors, and correspond to different sensitivity to anti-EGFR monoclonal antibodies (mAbs) [8], [9], [10].

*BRAF*, another component of the EGFR-RAS-RAF signal transduction pathway, encodes a RAS-regulated kinase that mediates cell growth, differentiation, apoptosis, and malignant transformation. Mutations of BRAF were found in several tumors, such as malignant melanoma, colorectal cancer, and so on [11]. To date, the presence of BRAF mutations has not been reported in penile SCC.

The RAS-association domain family 1 A (RASSF1A), a new RAS effector, is located on chromosome 3p21.3, a region frequently showing allelic loss in many cancers. Exogenous expression of RASSF1A decreases colony formation *in vitro* and tumor formation *in vivo* [12], [13], suggesting that it may be a tumor suppressor gene. Hypermethylation of CpG islands in the promoter region is the major mechanism for RASSF1A gene inactivation, which has been observed in many human cancers, including nasopharyngeal cancer, colorectal cancer, breast cancer and lung cancer. It was reported that RASSF1A functions as a tumor suppressor through RAS-mediated apoptosis. It was hypothesized that RASSF1A inactivation is closely related to RAS activation in human cancers, and therefore contributes to malignant transformation by inhibiting RAS-mediated apoptosis [13], [14], [15]. So far, the relationship between RASSF1A expression and K-RAS mutation has not been investigated in penile SCC.

To identify the potential role of EGFR-RAS-RAF signaling in penile SCC, we investigated four key members (EGFR expression, RASSF1A expression, K-RAS mutations, and BRAF mutations) of this pathway in 150 cases of penile SCC. We expect this information will provide us with guidance for using anti-EGFR mAbs as potential therapies for penile SCC. To our best knowledge, this is the first study to comprehensively investigate four essential genes at once in the EGFR-RAS-RAF signaling pathway in a large series of penile SCC patients.

## Materials and Methods

### Patients and Tissue Samples

Paraffin embedded tissues were collected from patients who underwent surgical resection for penile SCC at West China Hospital during January 2000 to May 2011. The pathological types of non-SCC and those treated with neoadjuvant chemotherapy and/or radiotherapy were excluded. Slides from enrolled samples were prepared for EGFR and RASSF1A immunohistochemical staining and used for genomic DNA extraction for KRAS and BRAF sequencing analysis. Clinicopathologic data of these patients were also collected. The study was approved by the Biomedical Ethics Committee of West China Hospital, Sichuan University, China. Written consent was given by the patients for their information to be stored in the hospital database and used for research.

### Immunohistochemistry Staining

Specimens were immunostained by standard labeled streptavidin-biotin protocol. Specifically, sections of 4 µm were mounted on silanized slides and allowed to dry overnight at 37°C. After deparaffinization and antigen retrieval, tissue sections were incubated with EGFR antibody (mouse monoclonal, 1∶300, Santa Cruz) or RASSF1A antibody (mouse monoclonal [3F3], 1∶30, Abcam) at 37°C for one hour, and then at 4°C for overnight. The sections were then incubated with biotinylated goat antimouse immunoglobulin G (Zymed Laboratories Inc, USA) and subsequently incubated with horseradish labeled streptavidin (Zymed Laboratories Inc, USA). 3, 3′-diaminobenzidine was used as chromogen and hematoxylin as counterstaining agent.

To evaluate EGFR and RASSF1A expression, five high power fields (×400) of tumor were randomly selected and 200 cells were counted per field. EGFR immunohistochemical evaluation was performed as previously described [16]. The percentage of labeled cells of EGFR expression was graded as follows: 0, no positive cells; 1+, 1–25% labeled tumor cells; 2+, 26–50% labeled tumor cells; 3+, 51–75% labeled tumor cells; 4+, >75% labeled tumor cells. Tumor tissues showing 3+ and 4+ were considered as EGFR over-expression. Nuclear and cytoplasmic reactivity for RASSF1A proteins was considered as positive or negative as described previously [17]. The percentage of labeled cells of RASSF1A expression was graded as follows: 0, no positive cells; 1+, 1–30% labeled tumor cells; 2++, >30% labeled tumor cells.

### Tumor DNA Extraction

Genomic DNA was extracted from paraffin embedded tissue as previously described [18]. Tissues consisting of at least 80% tumor cell content (reviewed by at least one experienced pathologist) were considered as eligible for DNA extraction. Tumor tissues were manually dissected from five consecutive 10 µm sections of the paraffin embedded tissues. The extracted tumor cells were collected into 190 μL digestion buffer (DNA tissue mini kit, Qiagen), and then treated with proteinase K overnight at 56°C. DNA purification was achieved using a nucleic acid robot device (BIO 101, Qiagen).

### Sequence Analysis

KRAS mutations at codons 12 and 13 and BRAF mutation at codon 600 were analyzed as previously described [18]. PCR amplification was done in a total volume of 20 μL containing 20 ng genomic DNA, 0·2 mM deoxynucleotide triphosphate, 0.5 units of Taq polymerase (HotStar Taq, Qiagen). The primer sets for codons 12 and 13 of the KRAS gene were 5′-AGGCCTGCTGAAAATGACTGAA-3′ (sense) and 5′-AAAGAATGGTCCTGCACCAG-3′ (antisense), flanking codons 12 and 13. The primer sets for codon 600 of the BRAF gene were 5′-TGCTTGCTCTGATAGGAAAATG-3′ (sense) and 5′-AGCCTCAATTCTTACCATCCA-3′ (antisense), flanking codon 600. For DNA sequencing, PCR was performed in a total volume of 10 µl containing the purified PCR products (20 to 50 ng), 1.6 pmol primer, 1 µl of BigDye terminator Mix, 1× adding buffer, and 0.1 units of Taq Polymerse. Cycle sequencing analysis of PCR fragments was done with the BigDye Terminator system (PE Biosystems) using amplification primers for bidirectional sequencing. The reaction products were analyzed on an ABI PRISM 3700 sequencer (PE Biosystems).

### Statistical Analysis

Statistical analysis was performed using SPSS 13.0 software. Correlations between EGFR expression, RASSF1A expression, KRAS mutation, BRAF mutation and the clinicopathological parameters were analyzed using the chi-square test for categorical variables. *P* values less than 0.05 were considered statistically significant.

## Results

### Patient Characteristics

A total of 150 patients were enrolled in this study. The mean age of the patients was 53.4 years (range from 24 to 83). Histological examination showed that 87 tumors were well differentiated SCC, 49 were moderately differentiated, and 14 were poorly differentiated. pT stage information was not available for 16 cases. For the patients with pT stages, there were 67 cases of T1, 59 of T2, 8 of T3, and no T4 cases. Among the 150 patients, 18 patients (12%) had lymph node metastases. The clinical characteristics of patients with examined tumors are listed in Table 1.

**Table 1:** The Clinicopathological Characteristics and EGFR Expression in the 150 Penile SCC.

	Total cases	Over-expression of EGFR			Positive expression of RASSF1A		
Factors	No.	No. (%)	χ^2^	*P*	No. (%)	χ^2^	*P*
Age							
<60	98	90(91.84)			3(3.06)		
≥60	52	48(92.31)	0.010	0.920	2(3.85)	0.065	0.799
Differentiation grade							
Poor-Moderate	63	60(95.24)	1.537	0.215	4(6.35)	2.985	0.084
Well	87	78(89.66)			1(1.15)		
pT stage *							
T1	67	59(88.06)	5.869	0.053	0(0)	5.836	0.054
T2	59	58(98.31)			4(6.78)		
T3	8	89(100)			1(14.29)		
Lymph node metastases							
Yes	18	17(94.44)			2(11.11)	3.668	0.055
No	132	121(91.67)	0.165	0.685	3(2.27)		

* pT stage information is not available for 16 cases and there were no T4 cases.

### EGFR Protein Expression and its Correlation with Clinicopathological Characteristics of Penile SCC

**Figure 1:**
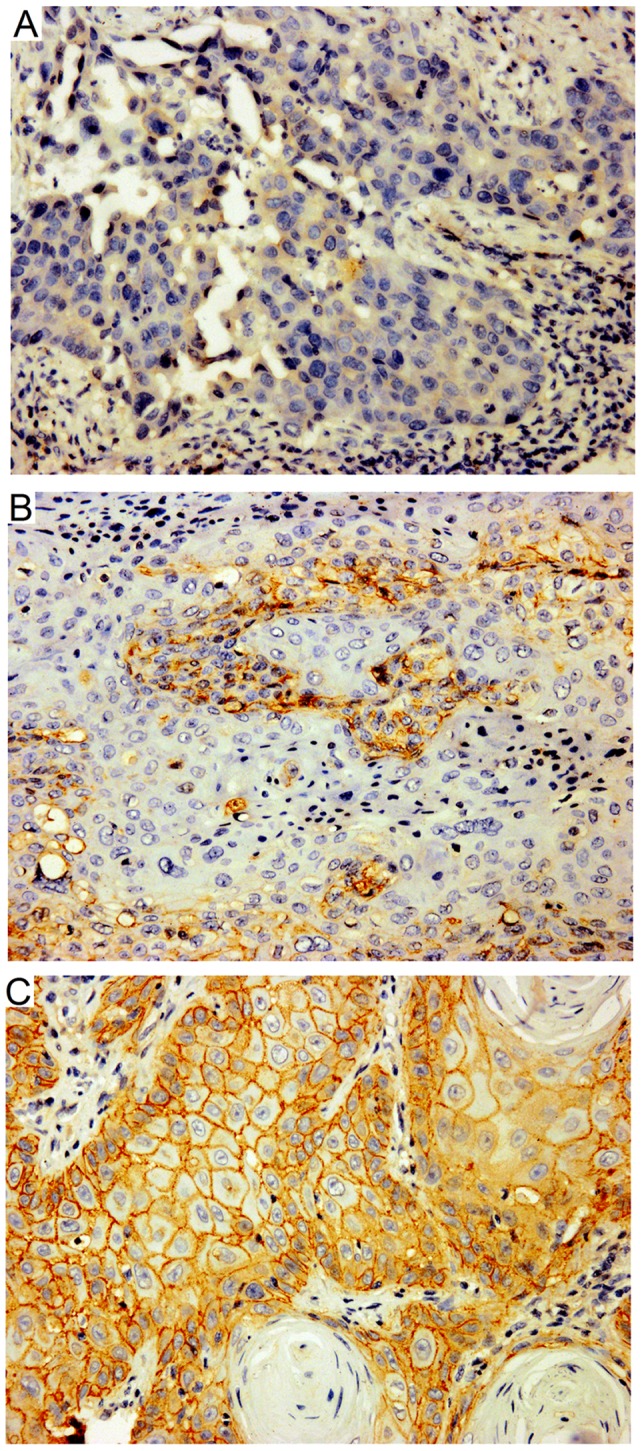
Expression of EGFR in penile SCC samples. All 150 samples were positive for EGFR expression, 5 cases (3.3%) were regarded as 1+, 7 cases (4.7%) were 2+, 138 cases were 3/4+ (92%). The representative figures of EGFR expression of low (A), moderate (B), and over-expression (C) were shown (EnVision, ×400).

EGFR protein expression was evaluated by immunohistochemistry in 150 patients. EGFR expression was positive in all of the 150 cases. Five cases (3.3%) were 1+, seven (4.7%) were 2+, 21 (14%) were 3+, 117 (78%) were 4+, as shown in Figure 1. The rate of EGFR over-expression was 92%. No significant correlation was observed between the EGFR expression and tumor grade (*P* = 0.215), pT stage (*P* = 0.053) or lymph node metastases (*P* = 0.685), as shown in Table 1.

### RASSF1A Protein Expression and its Correlation with Clinicopathological Characteristics of Penile SCC

**Figure 2:**
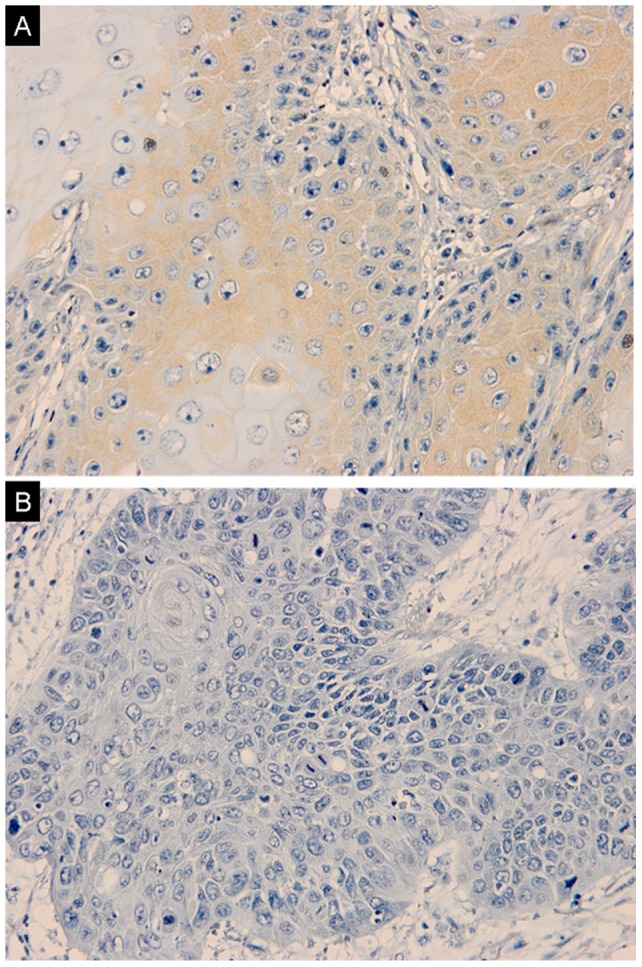
Expression of RASSF1A in penile SCC samples. Only 5 samples were positive for RASSF1A expression. The representative figures of RASSF1A expression of positive (A), negative (B) were shown (EnVision, ×400).

RASSF1A protein expression was also evaluated by immunohistochemistry in 150 patients. The patterns of RASSF1A proteins were mixed nuclear/cytoplasmic staining. As shown in Figure 2, positive expression of RASSF1A was found in 5 out of the 150 patients (3.33%). The rest of tumors (96.67%) did not have detectable expression of RASSF1A. No significant correlation was observed between the RASSF1A expression and tumor grade (*P = *0.084), pT stage (*P = *0.054) or lymph node metastases (*P* = 0.055), as shown in Table 1.

### Rare Incidence of KRAS Mutations in Penile SCC

DNA was extracted from 95 paraffin embedded penile SCC samples (63.3% of 150 patients). Most of the specimens that were not amendable for DNA extraction were from cases between 2000 and 2003. KRAS mutation analysis in codons 12 and 13 was successfully performed in 94/95 cases. Among the 94 evaluable cases, KRAS mutation was observed in only one sample (1.06%). A GGT > GAT transition was detected at codon 12 (G12D) in a 53-year-old patient with well differentiated tumor and no lymph node metastases. EGFR was overexpressed (4+) in this case. This result suggests that KRAS mutation is a rare event in penile SCC.

### No BRAF Mutation in Penile SCC

To detect BRAF mutation at codon 600, 95 DNA specimens were used for sequence analysis. BRAF mutation at codon 600 analysis was performed in 83/95 tumor specimens. No tumor was found to harbor a BRAF V600E point mutation among the 83 evaluable cases.

## Discussion

In our study, over-expressed EGFR was found in 92% of the penile SCC cases, and loss of RASSF1A protein expression was found in 96.67% of the cases. KRAS mutation analysis in codons 12 and 13 was performed in the tumor tissue of 94/150 patients, and BRAF mutation analysis in codon 600 was performed in 83/150 cases. KRAS mutations were observed in only one sample and no tumor was found to harbor a BRAF V600E point mutation. Due to the relatively small number of the mutational cases, we could not establish if KRAS mutation and BRAF mutation were associated with EGFR and RASSF1A expression, and the clinicopathological features of the patients.

The role of EGFR in the pathogenesis and progression of various malignant tumors has been extensively investigated. In our study, EGFR expression was positive in all specimens, and its over-expression rate was 92%. There was no correlation between the EGFR expression and tumor grade, pT stage or lymph node metastases. These results are consistent with the previous reports in several small-sample studies [19], [20], [21]. For example, Borgermann et al. showed that EGFR was highly expressed in 40 out 44 penile SCC cases [20]. The high expression of EGFR in penile SCC suggests that EGFR may play an important role in the pathogenesis of penile SCC.

Since the KRAS-BRAF pathway is a major EGFR-dependent signaling pathway, KRAS mutation may lead to anti-EGFR treatment failure. The characteristics of EGFR-RAS-RAF signaling pathway molecules of the penile SCC found in this study were similar to those of HNSCC. In HNSCC, EGFR was overexpressed in 70–90% of patients [22]. KRAS mutations were also rarely found in HNSCC [23], which may represent a good response to anti-EGFR mAbs, such as cetuximab or nimotuzumab [7], [9]. On the other hand, KRAS mutations occur in 40% of the patients with advanced CRC and it is also a powerful predictive biomarker for anti-EGFR therapy. These CRC patients who have mutation in codon 12 or 13 of KRAS gene are essentially insensitive to anti-EGFR mAbs and virtually derive no benefit from such therapy [8], [10]. Consistent with our data, Patiyan *et al* found only one KRAS mutations in 28 penile carcinomas [24]. Given the high expression of EGFR and rare KRAS mutations in penile SCC, anti-EGFR mAbs may represent an effective new treatment for this disease.

BRAF is another important mediator in the EGFR pathway. BRAF mutation rate varies among different types of tumors, predominantly in malignant melanoma, thyroid papillary cancer, and sporadic CRC. BRAF mutation was rarely found in HNSCC and lung cancer [23], [25]. BRAF mutation was once considered as a predictive biomarker, but recent studies have suggested that BRAF mutation was also a strong indicator of poor prognosis [25], [26]. In our study, no patient was found to harbor a BRAF V600E point mutation which suggested that BRAF mutation may not be an important event in the carcinogenesis of penile SCC.

RASSF1A, a potential tumor suppressor gene, is commonly expressed in normal tissues and silenced in numerous cancers through hypermethylation [13], [27]. Noaki *et al* showed that the frequency of RASSF1A methylation is 11.5% (3/26) in penile SCC [28]. In our study, we ventured to study the expression of RASSAF1A in penile SCC and found loss of RASSF1A expression in the vast majority of cases. This suggests that RASSF1A may be silenced by other mechanisms in addition to promoter CpG island hypermethylation in penile SCC. High frequency of loss of RASSF1A expression in penile SCC suggests that inactivation of RASSF1A may be associated with penile SCC pathogenesis. The exact involvement of RASSF1A in Ras signaling pathways is unclear. It was hypothesized that inactivation of RASSF1A is closely related to RAS activation in human cancers and RASSF1A methylation was an alternative way of affecting Ras signaling [14], [15], [29]. Relationships between expression of RASSF1A and K-RAS status had been studied in a few types of cancer. Several studies had shown an inverse correlation between RASSF1A silencing and K-ras activation [12], [30]. However, Kim *et al* reported that no association was found between *RASSF1A* methylation and *K-ras* mutation in 242 primary NSCLCs [12]. These different results may be attributed to tissue specificity and/or exposure to different environmental factors. In our study we found that K-*ras* mutation in penile SCC was rare, with only one case of K-ras mutation detected in a total of 94 cases, whereas loss of RASSF1A expression was a common event in penile SCC. Likewise, high frequency of RASSF1A inactivation is observed in tumor types with uncommon ras gene mutations, including small cell lung cancer, nasopharyngeal cancer and neuroendocrine pancreatic tumor [31], [32], [33] These observations suggest that the occurrence of frequent RASSF1A inactivation may exclude the necessity of K-ras activation to alter Ras signaling in carcinogenesis.

Collectively, our data strongly suggest that EGFR and RASSF1A may be important factors in the pathogenesis of penile SCC. Consequently, our findings suggest that agents such as anti-EGFR mAbs may be tested as a new treatment option for this disease. Prospective studies to examine the antitumor activities of anti-EGFR mAbs are necessary. However, due to the rarity of the disease, a large scale of clinical trial will be difficult to accomplish. Several case reports have demonstrated efficacy of cetuximab in in the treatment of advanced penile SCC. In a retrospective study involving 13 patients with advanced penile SCC, EGFR over-expression was detected in 77% of cases. These patients received anti-EGFR targeted therapies including erlotinib (one patient), cetuximab (three patients) and cetuximab combined with cisplatin-based chemotherapy (nine patients). These treatments achieved a median time to progression of 3.2 months and a median overall survival of 9.8 months, suggesting that anti-EGFR targeted therapies have favorable efficacy in these patients [19].

To our best knowledge, we are the first to comprehensively investigate the EGFR-RAS-RAF signaling pathway in a large set of patients with penile SCC. Of note, some genomic DNA samples were not able to be extracted from paraffin embedded tissues. This might be ascribed to DNA-tissue protein cross-links and/or nucleic acid fragmentation due to aging of the specimen or the pH of the fixative [34]. Nonetheless, KRAS and BRAF mutations analysis was finally performed in 94/150 (62.67%) and 83/150 (55.33%) of tumors, respectively. Mutation analysis was completed for more than half of the cases, and the absolute majorities of them had no mutation. This makes us believe that our current results are still valuable for evaluation of the status of EGFR-RAS-RAF signaling and supports the potential use of anti-EGFR mAbs in the treatment of penile SCC. The analysis of some downstream proteins (such as ERK and pERK) of EGFR pathway is also very important for us to understand the mechanisms of pathogenesis of penile SCC. However, our study is a retrospective analysis which could not perform further mechanistic investigation. Thus, we are planning to proposal a prospective study to get more information about this disease.

## Conclusions

Our data demonstrated that the majority cases of penile SCC are associated with over-expression of EGFR and loss of expression of RASSF1A, a potential tumor suppressor. KRAS and BRAF mutations are extremely rare events in penile SCC. These findings suggest that blockade of EGFR signaling with anti-EGFR mAbs might be a biologically rational approach for the treatment of penile SCC.
